# High Accuracy Weigh-In-Motion Systems for Direct Enforcement

**DOI:** 10.3390/s21238046

**Published:** 2021-12-01

**Authors:** Piotr Burnos, Janusz Gajda, Ryszard Sroka, Monika Wasilewska, Cezary Dolega

**Affiliations:** 1 Department of Measurement and Electronics, AGH University of Science and Technology, Al. A. Mickiewicza 30, 30-059 Krakow, Poland; jgajda@agh.edu.pl (J.G.); rysieks@agh.edu.pl (R.S.); 2 Department of Acoustics, Multimedia and Signal Processing, Wroclaw University of Science and Technology, Wybrzeze Wyspianskiego 27, 30-370 Wroclaw, Poland; monika.wasilewska@pwr.edu.pl; 3 Neurosoft, Sp. z o. o., Zyczliwa 8, 53-030 Wroclaw, Poland; cezary.dolega@neurosoft.pl

**Keywords:** Weigh-In-Motion, direct enforcement, e-WIM systems, high accuracy WIM systems

## Abstract

In many countries, work is being conducted to introduce Weigh-In-Motion (WIM) systems intended for continuous and automatic control of gross vehicle weight. Such systems are also called WIM systems for direct enforcement (e-WIM). The achievement of introducing e-WIM systems is conditional on ensuring constant, known, and high-accuracy dynamic weighing of vehicles. WIM systems weigh moving vehicles, and on this basis, they estimate static parameters, i.e., static axle load and gross vehicle weight. The design and principle of operation of WIM systems result in their high sensitivity to many disturbing factors, including climatic factors. As a result, weighing accuracy fluctuates during system operation, even in the short term. The article presents practical aspects related to the identification of factors disturbing measurement in WIM systems as well as methods of controlling, improving and stabilizing the accuracy of weighing results. Achieving constant high accuracy in weighing vehicles in WIM systems is a prerequisite for their use in the direct enforcement mode. The research results presented in this paper are a step towards this goal.

## 1. Introduction

Due to the design features of Weigh-In-Motion (WIM) systems, they are sensitive to the influence of many factors which can disturb the measurement. The specific design of such systems is a result of the load sensors being installed directly in the road, which is part of the measuring system [1]. The influence of the temperature of the pavement appears to be the most important of these disturbing factors. Fluctuations in temperature change the pavement parameters, such as the stiffness modulus or vertical deflection, which in turn varies the accuracy of the weighing results. These changes depend on the technology used in the construction of the road surface as well as the construction of the load sensors and the method of their installation. In addition to temperature, the weighing result is influenced by the direction and strength of the wind, water film, the icing on the road surface, and vehicle speed. Numerous scientific reports point to this important feature of WIM systems [2,3,4]. The variability of the above-mentioned factors change the accuracy of the weighing results. The observed fluctuations take place in a single day or even over just a few hours. In practice, this means that the accuracy of the WIM system may be subject to uncontrolled change within as little as a few hours after the completion of a standard calibration procedure.

The great importance of WIM systems for traffic and transport engineering expands the area of their application. As a consequence, there is a need to study the properties of WIM systems operated in various climatic conditions. The results of such studies are presented, for example, in the works [5,6,7,8,9]. Manuals addressed to users and suppliers of WIM systems are also being developed [10]. The work [5] concerns the analysis of the influence of winter weather conditions on the accuracy of weighing in WIM systems. The aim of the research was to build a model describing the impact of winter operating conditions of WIM systems on their operation. The research used the results of measurements collected from five different WIM systems over a period of 6 years. It has been shown that the built model can be transferred between WIM systems operating in different locations. In [8], the subject of the assessment was the variability of such a model as a function of time. As a result, it was confirmed that the developed model was correct in the following years of its application. The work [6] discusses the results of periodic assessment of the accuracy of measurements in WIM systems operated in winter conditions. The accuracy of the systems was verified on the basis of periodic calibration, which included both the measurement of the length and speed of the vehicle, as well as gross vehicle weight and axle loads. The assessment of the accuracy of weighing vehicles in the WIM system equipped with piezoelectric load sensors is presented in [7]. The evaluation was carried out on the basis of the weighing results of 5-axle tractor semi-trailer trucks. In [9], the subject of the tests is a new capacitive flexible load sensor intended for use in WIM systems. Based on the research, it was found that the relative measurement error is less than 10%. The handbook [10] provides good practice recommendations for aspects of accuracy, quality assurance, sites, installation, and calibration. The basis for the formulation of these recommendations was the experience resulting from the long-term use of various WIM systems. In particular, WIM systems equipped with a bending plate, piezoelectric sensors and a load cell were investigated.

As the requirements for Weigh-In-Motion systems for direct enforcement (e-WIM) [11,12] include, first of all, high and stable accuracy of weighing results, it is reasonable to research and identify factors disturbing measurement and develop methods to reduce their impact. This was the primary motivation for the authors of this paper to undertake this research. The study was carried out in 2017–2018 on two WIM systems installed in Poland, equipped with Kistler quartz load sensors. One of the goals of this study was to determine the factors influencing the accuracy of the WIM system, quantify this impact, and build mathematical models of these interactions. In our research, we used the characteristic vehicles method, which allows for continuous assessment of the metrological properties of the WIM system. This paper presents the most critical conclusions resulting from the research conducted. We discuss ways of identifying the non-stationarity of WIM systems and propose strategies to improve the accuracy of weighing results and maintain constant accuracy over a long time horizon. This issue is crucial for e-WIM systems.

## 2. Measurement Data

**Figure 1:**
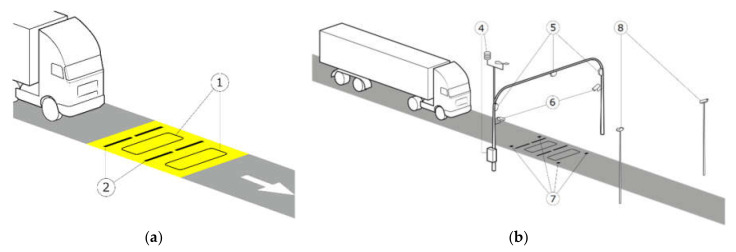
Diagram of sensor installation in WIM systems: (**a**) basic system configuration, (**b**) extensive system configuration, where: 1—inductive loops, 2—linear pressure sensors, 4—weather station, 5—3D scanners, 6—side cameras, 7—integrated temperature and surface condition sensors, 8—viewing cameras.

The analysis of the impact of factors disturbing the result of weighing vehicles in motion is based on data collected from two measurement systems located in Poland, in Kochanow (national road 79) and Grodziec (national road 46). In both of the WIM systems studied, quartz load sensors and induction loops serving as vehicle detectors were installed. The same sensor arrangement scheme was used in both systems (Figure 1a).

For this study, both WIM systems were equipped with additional sensors for measuring atmospheric parameters (Figure 1b). In the case of the WIM system installed in Kochanow, the station was also equipped with surface temperature sensors seated at a depth of 50 mm. In Grodziec, integrated sensors were built into the surface which allowed for two-point temperature measurement: on the road surface (0 mm) and at a depth of 50 mm, i.e., the depth at which the weighing sensors are mounted. The system in Grodziec was also equipped with sensors and software that made it possible to associate the measurement results with photos of passing vehicles. Additional sensors made it possible to measure the condition of the surface (humidity, salinity). To assess the influence of factors such as air temperature and humidity, wind direction, or precipitation intensity on the accuracy of vehicle weighing results, a weather station was installed next to the measuring field. The station also made it possible to measure the ground temperature at a depth of 300 mm. In order to verify the correctness of the weighed vehicle’s passage through the WIM system, side cameras and an overview camera were linked to the system. Using this additional data, the software recognized the class and model of the vehicle. Data from both WIM systems were collected for one year. Measurements in these locations were carried out independently of each other and at different times. The number of records stored in the databases is shown in Table 1.

## 3. Characteristic Vehicle Method

In the process of calibrating WIM systems, the most common method is by using pre-weighed vehicles which act as a standard. This involves using trucks belonging to selected classes with known gross vehicle weight and individual axle load. These vehicles pass through the weighing station many times. Reference values of gross vehicle weight and axle load are determined on accurate static scales or LS-WIM scales with a legalization certificate. Comparing of the reference values with the results obtained from the WIM system allows for the determination of weighing errors and calibration. The scenario for implementing such a procedure is described, for example, in [13]. However, the pre-weighted vehicle method does not allow for continuous monitoring of the accuracy of WIM system, a prerequisite for the implementation of e-WIM systems. In addition, this method also has other disadvantages, namely:

- it is necessary to rent several trucks with different axle configurations, which generates considerable costs,
- precise determination of reference values is troublesome and requires a special station and certified platform and static or slow-speed scales.
- the measurements on WIM site should be carried out for different levels of vehicle loading, i.e., for different values of the gross vehicle weight, and the number of passes should be at least 50–60. In addition, these passes must be made at different speeds. As a result, such an experiment is time-consuming and may take 6–8 h.
- in practice, thus it is not possible to conduct such measurements for a period of time sufficiently long to determine the long-term fluctuations of the WIM system errors. The pre-weighed vehicle method allows to determine only the accuracy of the system under the conditions of its use prevailing over time.
- for apparent reasons, repeating such a procedure is not possible more often than once every few months.

The characteristic vehicles method is not subject to these disadvantages. It enables continuous control of the accuracy of the WIM system.

In relation to the pre-weighed vehicle method, in the characteristic vehicle method, instead of the accurate weighing results, the mean value of the first axle load of a certain group of vehicles is used as the reference value. This mean value will be denoted as $\bar{w}$ and called characteristic value.

The method is based on the use of heavy goods vehicles, selected from normal road traffic. As a result of studies conducted, it has been shown that the class of 5-axle trucks with a strictly defined structure is characterized by a stable static load of the first axle. The load on this axle is poorly correlated with the gross vehicle weight and is characterized by low variability. Therefore, this axle load can act as a reference quantity, and it’s mean value as a reference value—called characteristic value. The characteristic value was determined in two stages:

Stage I—Selection of the class of the characteristic vehicles—analysis of traffic data from various locations of WIM systems.

Stage II—Determination of the characteristic value $\bar{w}$—data analysis from accurate static and slow-speed scales with legalization certificate.

**Figure 2:**
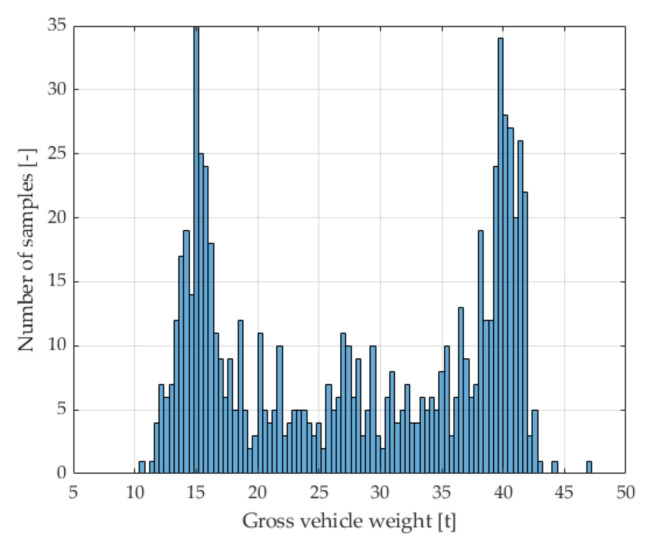
Distribution of the gross vehicle weight of 756 trucks (2-axle tractor + 3-axle semitrailer) for data from the Kochanow system.

In the first stage, measurement data from several WIM systems installed in Poland were analyzed. The aim was to isolate a class of characteristic vehicles. The type structure of traffic was assessed, as well as the number of vehicles in individual vehicle classes in selected locations. The determination of the number of vehicles was important, because for the long-term assessment of the accuracy of WIM systems, it is necessary that runs of characteristic vehicles were performed with high frequency, i.e., at least 100 times a day. Then, a statistical analysis of the weighing results was carried out in order to estimate the parameters of the axle load probability distributions of vehicles belonging to different classes. It was shown that the load of the first axle of trucks of the 2-axle tractor + 3-axle semitrailer class has the lowest random variability and is the least correlated with the gross weight of the vehicle. Selected elements of the standardized covariance matrix determined for the results of measuring the gross vehicle weight and the axle load of vehicles belonging to this class are presented in Table 2. The presented values of the elements of the covariance matrix contain information about the correlation of individual quantities with each other: the value 0 means no correlation and the value 1—complete correlation. The distribution of the total weight is shown in Figure 2.

**Figure 3:**
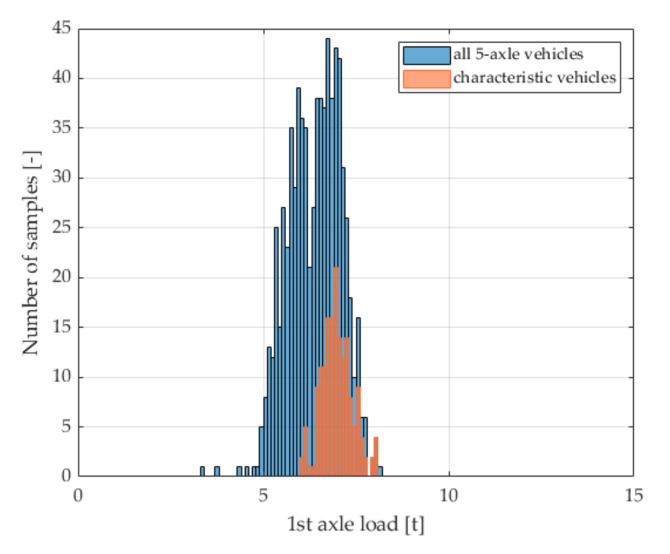
Histograms of the first axle load of all trucks 2-axle tractor + 3-axle semitrailer and vehicles selected as characteristic vehicles, for data from the Kochanow system.

The result of the research was the determination of a group of filters that allowed the selection of a sub-class of the characteristic vehicles with specific features only, chosen from 5-axle trucks. In this sub-class constituting a set of characteristic vehicles, we obtained an even lower dependence of the load of the first vehicle axle on the gross vehicle weight. This is confirmed by the elements of the normalized covariance matrix presented in Table 3. Importantly, in the set of characteristic vehicles, we also observed a reduction in the standard deviation of the mean value of the load of the first axle of the selected vehicle class from 10.98% to 6.70% (Figure 3).

Since the first axle load of the reference vehicles is statistically stable, its mean value $\bar{w}$, called characteristic value, can be used in calibration and to assess the accuracy of WIM systems. The characteristic value $\bar{w}$ serves as a reference and in the evaluation of the WIM system errors it is compared with the results from the assessed WIM system. This results in the frequency of assessing the accuracy of the system or its auto-calibration equal to the frequency of occurrence of characteristic vehicles (at least 100 times a day).

The characteristic value $\bar{w}$ was determined in the second stage of the research, based on the analysis of data obtained as a result of the precise weighing of characteristic vehicles. In total, we analysed several thousand weighing results of such vehicles. Weighing was carried out on static scales with a certificate of legalization, ensuring accuracy of within 1 or 2%. The data sets on the basis of which the mean value $\bar{w}$ of the first axle load of the characteristic vehicles was determined came from two separate periods, thus enabling the mutual verification of the correctness of the obtained results of the experiment. 

The characteristic value is determined once, usually for the area of a given country. Thus, it is used as a reference for the evaluation of many WIM systems in a given area.

The research methodology for assessing the accuracy of the WIM system with the use of a characteristic value will be described later in this paper. 

## 4. Factors Affecting Weighing Accuracy in WIM Systems

The idea behind WIM systems is relatively simple: the dynamic load exerted by the wheels of successive axles of a vehicle rolling over a load sensor installed in the road pavement is directly measured. On this basis, the value of the static load of each axle is estimated, and their sum is an estimate of gross vehicle weight. Various disturbing factors influence the measurement and, consequently, the accuracy of the estimated values of load and gross vehicle weight. The current state of knowledge on the sensitivity of WIM systems to disturbing factors shows that these factors can be divided into three main categories [1,2,14,15]:

a. Factors related to the vehicle to be weighed include:
  - Vertical fluctuations of the vehicle affecting the amplitude of the dynamic component of the load (depending on the vehicle speed, suspension parameters, gross vehicle weight, tyre type, tyre pressure, axle configuration),
  - Speed and speed change of the vehicle while passing through the WIM system (depending on the human factor, terrain, signposting, and traffic conditions),
  - Airlift effect (depending on the wind speed, its direction in concerning to the direction of travel, vehicle speed, and vehicle silhouette).
b. Road surface factors include:
  - Road quality (depending on road geometry, its vertical profile, quality of materials used, and quality of workmanship),
  - Spatial repeatability of the axle load (depending on the vertical profile of the road on the path through the WIM station),
  - Change of pavement parameters under the influence of temperature changes and axle load of a moving vehicle,
c. Factors related to the WIM system include:
  - The number and technology of the load sensors used,
  - The method of their assembly and arrangement along with the WIM station,
  - Speed measurement accuracy,
  - Accuracy and frequency of system calibration.

By analyzing the factors as mentioned earlier, it is easy to identify those which are influenced by the system designer and those which, although measurable, cannot be controlled (e.g., stabilized). This leads to four strategies for reducing the impact of vehicle weighing disturbing factors in WIM systems.

The elimination strategy is to design the system so that a given factor has no or minimal effect on the measurement. For example, concerning to the selection of the location of the WIM system, particular criteria are used to determine the unique geometric parameters of the road on which a WIM station can be installed [13]. Similarly, disturbing factors such as electrical power fluctuations or the influence of the electromagnetic field on the measuring equipment and axle load sensors are eliminated. 

When a factor cannot be eliminated but is measurable and a mathematical model is known, a compensation strategy can be used. This consists of using a mathematical model of the influence of a given factor and the correction of the weighing results on an ongoing basis, proportional to the intensity of the influencing factor. An example of quantities whose influence can be compensated in this way is the pavement temperature and the speed of the weighed vehicle.

If the given factor is measurable but the mathematical model of its influence on the weighing results is not known, a strategy of rejecting the measurement can be used. For example, if the system is able to detect heavy rainfall and film formation on the pavement, but the model for this impact is not known, it is not possible to apply a compensation strategy. In such a case, the measurement results should be rejected. 

If, for any reason, none of the above-mentioned strategies can be used, because the system cannot detect or measure the intensity of the disturbing factors or the mathematical models of their interactions are not known, then the auto-calibration strategy [16,17] can be used. This is based on the application of an algorithm that uses characteristic vehicles and a known mean value $\bar{w}$ of characteristic quantity to calibrate the WIM system continuously. This calibration is performed with the frequency of the passes of the characteristic vehicles. Thus, the calibration coefficients of the WIM system keep up with the changes of influencing factors.

## 5. Research Methodology

The determined characteristic value $\bar{w}$ can be used as a reference to evaluate the accuracy of the system and its auto-calibration. Knowing the value of $\bar{w}$ allows for the comparing of the current weighing results of the characteristic vehicles from the WIM system with a value of $\bar{w}$. This opens the way for continuous monitoring of the accuracy of the WIM system, as well as its auto-calibration, without the need to use the pre-weighted vehicle method. By examining the accuracy of the system, one can calculate a single numerical measure, for example a relative error, or determine the accuracy characteristics showing the influence of a given measurement disturbing factor on the value of this error. 

To determine the accuracy of the WIM system under given conditions, it is enough to collect a statistically significant population of weighing results for characteristic vehicles from WIM system, calculate the mean value of the first axle load of these vehicles, and compare it with the characteristic value $\bar{w}$.

**Figure 4:**
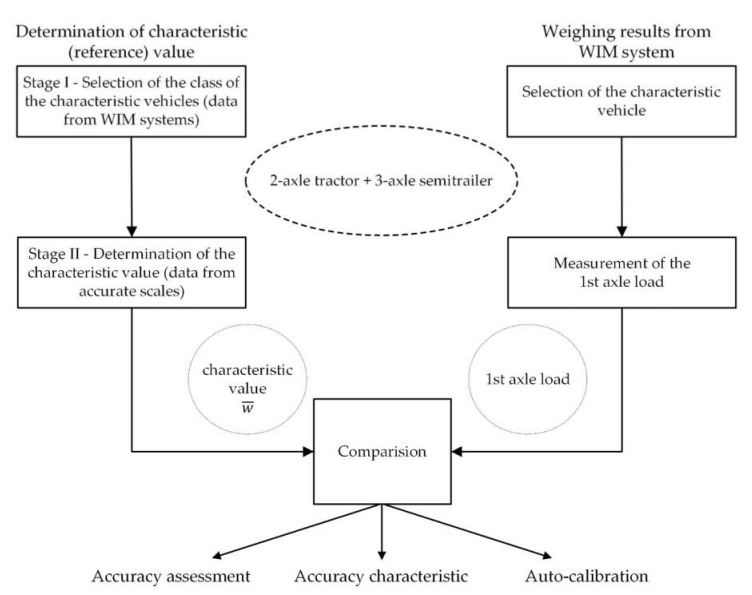
The diagram of the methodology for assessing the accuracy and auto-calibration.

Determining the accuracy characteristic requires the collection of a more significant number of characteristic vehicle weighing results in the WIM system, in a wide range of changes of the disturbing factor (e.g., temperature, wind direction, etc.). Therefore, such measurements should be carried out over a more extended time, during which the values of disturbing factors change significantly (preferably covering the three seasons, e.g., summer, fall and winter). Then, for each value of the disturbing factor and with the constant value of the remaining disturbing factors, the weighing results of the first axle are averaged. The mean value determined in this way is compared with the characteristic value $\bar{w}.$ As a result, this leads to the determination of the accuracy characteristic illustrating the dependence of the WIM system error on the intensity of the disturbing factor. Measurement data were approximated by second order model. In this way, the following models of the influence of the surface temperature, the speed of the weighed vehicle and the wind direction were determined. The diagram of the methodology for assessing the accuracy and auto-calibration is shown in the Figure 4.

### Auto-Calibration Method

The autocalibration method was first proposed by Stanczyk [18], when introducing the so-called autocalibration algorithm of WIM systems. It was developed and improved by Burnos, and the results of his work were published for the first time in 2009 [19] and then in 2012 [17] and 2020 [20].

Auto-calibration of the WIM system consists in tuning the slope of the static characteristic of the system (gain) in such a way as to obtain the correct result of the measurement of the characteristic quantity, under current operating conditions. The implementation of this idea requires the periodic presence of characteristic vehicles. In the case of WIM systems, the role of the reference value is played by the characteristic value determined on the basis of the previously described operation in Section 3.

The recursive least squares algorithm (RLS) [21] known from the literature was used to tune the calibration coefficient *S*, the forgetting coefficient of which was selected to minimize the weighting error (1).

(1) $$S_{n}=S_{n-1}+K_{n}\left(\bar{w}-{\mathrm{Wd}}_{n}\cdot S_{n-1}\right),$$

(2) $$b_{n}=1./\left({\mathrm{Wd}}_{n}\cdot P_{n-1}\cdot {\mathrm{Wd}}_{n}+\lambda \right),$$

(3) $$K_{n}=P_{n-1}\cdot {\mathrm{Wd}}_{n}\cdot b_{n},$$

(4) $$P_{n}=\left(P_{n-1}-K_{n}\cdot {\mathrm{Wd}}_{n}\cdot P_{n-1}\right)/\lambda ,$$

where:

$\bar{w}$—characteristic value,
λ—forgetting factor with values in the range (0–1),
${\mathrm{Wd}}_{n}$—the result of dynamic weighing (measurement of the reference value) of the *n*-th characteristic vehicle at the WIM station,
*n*—number of the subsequent characteristic vehicle that has driven through the WIM station,
$S_{n}$—estimate of the WIM system calibration coefficient determined in the *n*-th iteration.

The implementation of this algorithm allows for the continuous update of the slope $S_{n}$ of the static characteristics of the WIM system. Alteration of this slope is possible after each passage of a characteristic vehicle through the calibrated WIM system. The dynamics (speed) of tuning of this parameter is determined by the assumed value of the forgetting factor λ. Detailed research results of this algorithm were published in [17].

## 6. Analysis of Measurement Data from WIM Systems

The quantitative assessment of the influence of some of the above-mentioned factors on the accuracy of vehicle weighing in WIM systems was the aim of the authors’ earlier works [1,2,17]. The current study investigated in detail, among others, the effects of pavement temperature, the speed of the weighed vehicle, and the direction of the wind. The study of the impact of speed and temperature was aimed at verifying the results obtained in previous studies. The study of the influence of wind direction is a new element, expanding the knowledge of the metrological properties of the WIM system.

The above-mentioned disturbing factors for vehicle weighing create a multidimensional map of interactions and influence each other. It is therefore important to determine the cross-correlation between these factors as well as between them and the results of the weighing of characteristic vehicles. The figures below show the correlation between the air temperature and the surface temperature, and between the surface temperature and the weighing results of the characteristic vehicles.

**Figure 5:**
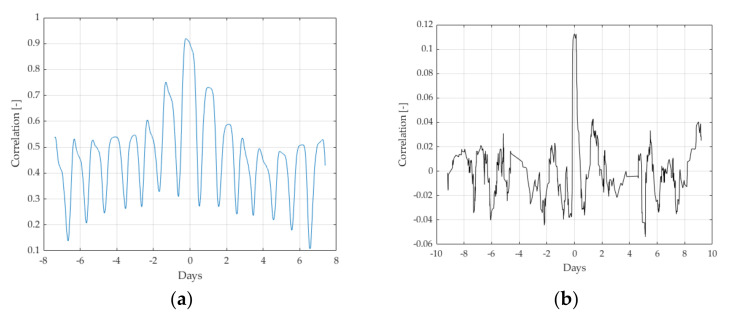
(**a**) Correlation function between the air temperature and the surface temperature measured at a depth of 50 mm (**b**) Cross-correlation functions between the surface temperature measured at a depth of 50 mm, and the result of weighing the first axle of vehicles characteristic for the data obtained from the system in Kochanow.

Figure 5a shows a significant correlation between the air temperature and the surface temperature (maximum value> 0.9). In addition, the impact of the dynamics of the heat transport phenomenon along the road is visible. The maximum correlation of both values occurs for approx. 2 h, which means that after 2 h from the occurrence of the air temperature change, the pavement temperature changes. The characteristics presented in Figure 5b show the correlation of weighing results with the surface temperature. The maximum value of this correlation (0.115) seems to be relatively small. However, in the context of the discussion of WIM systems operating in direct enforcement mode, this is a significant value. As can be seen from the characteristics presented in Figure 5 even such a small correlation causes a weighing error of approximately 1–3 percent.

The obtained results lead to an important conclusion: when implementing the compensation strategy, the pavement temperature measurement results should be used, not the air temperature measurement. It should also be emphasized that the latter solution is normally implemented by weather stations cooperating with WIM systems. Similarly, the correlation between other influencing quantities and their influence on the accuracy of weighing the first axis of characteristic vehicles was assessed. The obtained results allowed for the determination of a set of values that have a significant impact on the operation and accuracy of the WIM system. These are road surface temperature, vehicle speed, and wind direction.

**Figure 6:**
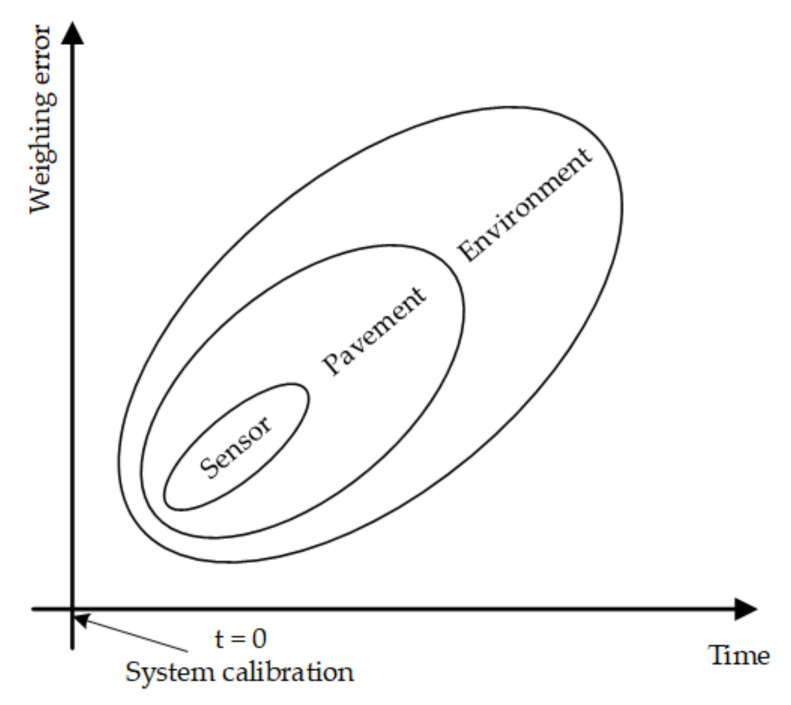
Weighing Error Components and Time of Occurrence.

The relationship between the factors as mentioned above causing the weighing error and the time in which their impact on the WIM system becomes apparent is shown in Figure 6.

The parameters and chosen characteristics of the load sensors are specified by the manufacturer and should be taken into account at the stage of designing the WIM weighing station. They are known a priori and have relatively the most minor contribution to the balance of weighing error in WIM systems (provided we do not take into account the increasingly infrequently used polymer sensors). Secondly, the road surface properties should be considered. The material from which it is made (asphalt or concrete), its evenness and geometry should be known at the stage of selecting the location of the WIM system. A winding road with high unevenness causes vertical bouncing of the vehicle and hopping of its wheels, which increase the variability of the vehicle axle load on the ground (dynamic component), and this in turn increases the error in the estimation of the static component of this load. By following the recommendations concerning the selection of the system location [13], the influence of this factor on the weighing error can also be minimized.

### 6.1. Influence of Temperature

**Figure 7:**
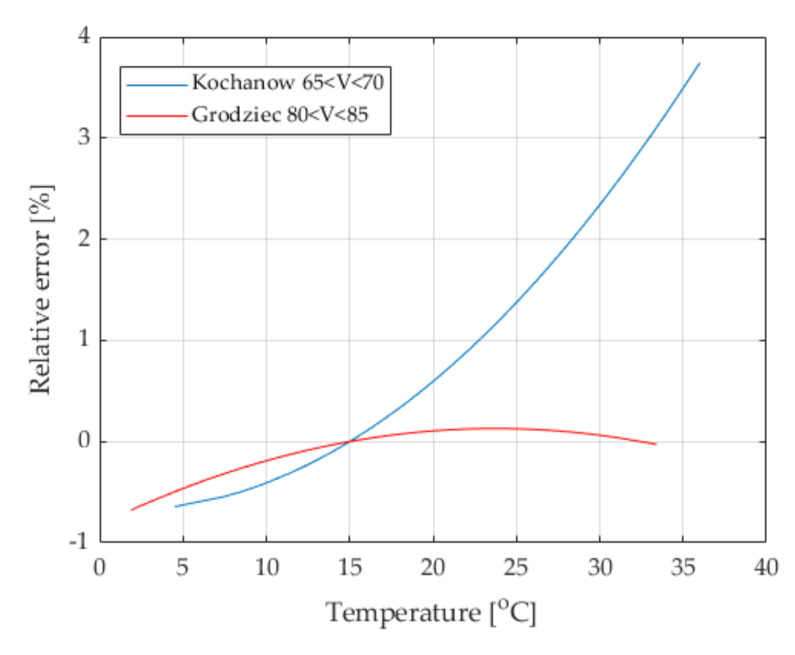
Comparison of temperature characteristics for the locations Grodziec and Kochanow. The speed of the weighed vehicles was in the range of 80–85 km/h (Grodziec) and 65–70 km/h (Kochanow).

**Figure 8:**
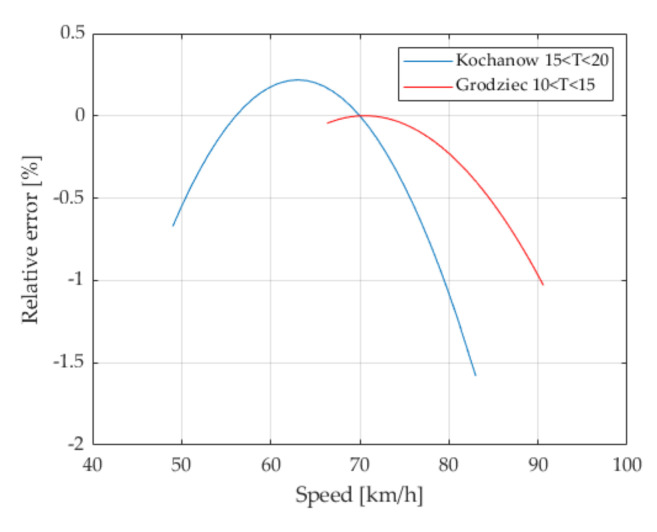
Influence of the speed of the weighed vehicle on the error of its weighing, for the locations Grodziec and Kochanow. The surface temperature was in the range 10–15 °C (Grodziec) and 15–20 °C (Kochanow).

**Figure 9:**
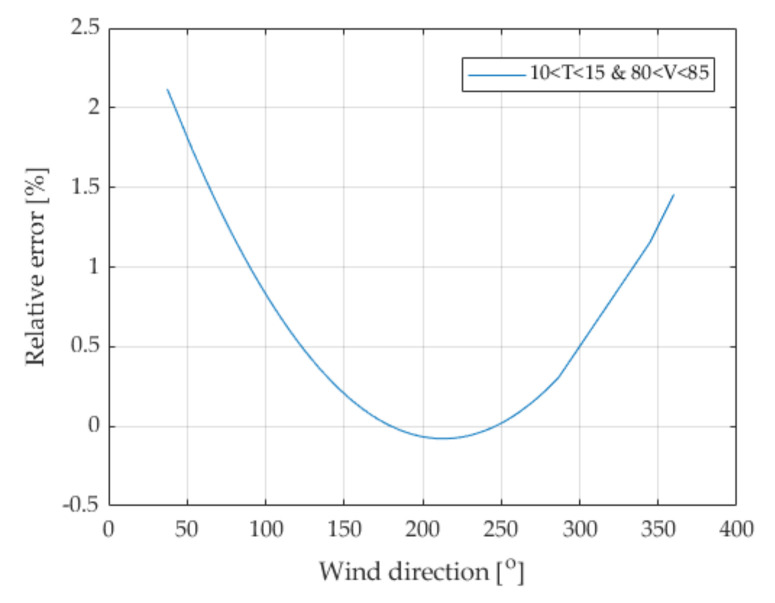
Influence of wind direction on weighing error for the Grodziec location. The surface temperature was in the range (10–15 °C) and the speed of the weighed vehicles was in the range of (80–85 km/h).

The influence of environmental factors on errors of the WIM system is complex to determine at the system construction stage, as this depends on changing and unpredictable weather conditions. Figure 7, Figure 8 and Figure 9 show the characteristics illustrating the influence of environmental factors on weighing accuracy in WIM systems. The measurement data used to determine these characteristics were selected such that only one influencing quantity varied within wide limits, while the others were within a narrow range of values. This allowed for the selective examination of the influence of only one selected factor on the weighing results. Measurement data were approximated by second order model. The presented characteristics illustrate the dependence of the relative vehicle weighing error on the intensity of the selected influencing factor. These were determined on the basis of measurement data obtained from the two WIM systems located in Grodziec and Kochanow.

The presented characteristics allow for the conclusion that the intensity of the influence of pavement temperature strongly depends primarily on its type (system location), because in both locations the same kind of sensors with the same thermal properties was used. It can also be seen that this influence cannot under any circumstances be ignored. The influence of temperature is especially significant in the Kochanow location. There, changes of temperature within the range of 30 °C cause a weighing error exceeding 5%. This impact can be limited by using a compensation or auto-calibration strategy.

### 6.2. Speed Effect

The analysis shows that the correlation between the weighing result and vehicle speed is negative. This means that an increase in the speed of the weighed vehicle reduces the weighing result (negative measurement error). The observed phenomenon may be explained by the airlift effect, especially since the subject of the measurement was the load of the first axle of the characteristic vehicle. This is confirmed by the characteristics shown in Figure 8.

The obtained results show that when weighing heavy goods vehicles in WIM systems, an axle weighing error of 1% should be accounted for, caused by the different speeds of the weighed vehicles passing through the WIM station, within the range (50 km/h–80 km/h). This effect is additive to the temperature effect and must also be compensated for in the highest precision systems. 

### 6.3. Influence of Wind Direction

A similar methodology was used for the quantitative assessment of the impact of the third factor disturbing weighing in the WIM system, namely the wind. Wind direction measurement was performed only at the WIM system located in Grodziec.

The symmetrical course of the characteristic presented in Figure 9 positively verifies the correctness of the measurements of this parameter and the performed calculations. Our hypothesis about the influence of wind on the weighing result for large vehicles was confirmed. Wind blowing from a certain direction can cause an airlift effect and mass transfer from one axle of the vehicle to another. As can be seen from the presented characteristics, this phenomenon may cause a change in the vehicle weighing result by up to 2%.

## 7. Practical Application of the Research Results

In practice, a certain version of the described autocalibration algorithm has been implemented, which we have called statistical calibration. This method consists in calibrating the system periodically following the customer’s requirements (e.g., once a month or every six months) and continuously, i.e., after each pass of a characteristic vehicle. This allows for the control of the operation of this method in the actual operating conditions of the WIM system. Such caution is justified in the initial stage of the implementation of the auto-calibration method and allows the knowledge on this subject obtained through modelling and simulations to be expanded and verified. The results of the statistical calibration are used to periodically check the correctness of the WIM system operation and possible change of their calibration coefficients S. The calculated correction of the calibration coefficients is not automatically applied in the system—the operator always decide this. In order to apply the method of statistical calibration and increase its accuracy, a statistical standard of the characteristic value has been proposed, calculated on the basis of the results of dynamic weighing. In this case, the reference value was calculated on the basis of measurements from WIM systems that were previously calibrated using the method of pre-weighed vehicles. The implementation of this method involved systems installed in Poland and Germany. All WIM systems tested in this way, after applying the new standard and introducing corrections of the calibration coefficient S, and before the next calibration with pre-weighed vehicles, achieved the accuracy of the weighing system at the level A5 or B7 (+) according to the COST323 standard [14]. 

## 8. Summary

Identifying the causes of changes in the accuracy of weighing in WIM systems has become the primary motivation for searching for solutions that would stabilize the metrological properties of these systems. The proposed method is based on auto-calibration of the WIM system, carried out continuously or periodically at the operator’s request (statistical calibration). The condition for the practical implementation of this method was to build a statistical load model. Our research as well as design and experimental work have led to the development of an effective method of building such a model. As a result of the undertaken research, design, and experimental work, it has been shown that it is possible to build WIM systems that maintain high and constant accuracy. Increasing the accuracy of weighing vehicles in WIM systems brings measurable effects. Regardless of whether WIM systems are used as pre-selection systems (currently, this is the most popular application of WIM systems) or they function as systems for direct enforcement, an improvement in weighing accuracy increases the efficiency of vehicle weight control and thus the effectiveness of efforts to eliminate overloaded vehicles from road traffic. This, in turn, translates directly into the protection of road infrastructure, increasing the safety of road users, environmental protection, and ensuring the conditions of fair competition in transport.

## Figures and Tables

**Table 1:** Number of weighing results from HS-WIM systems stored in databases.

Location of the WIM System	The Number of Weighed Vehicles
Grodziec	223,143
Kochanow	213,545

**Table 2:** Normalized covariance matrix for all trucks 2-axle tractor + 3-axle semitrailer based on data from the Kochanow system.

	Load 1 Axle	Load 2 Axle	Load 3 Axle	Load 4 Axle	Load 5 Axle
Gross vehicle weight	0.776	0.942	0.983	0.985	0.984

**Table 3:** Normalized covariance matrix for characteristic vehicles, determined on the basis of data from the Kochanow system.

	Load 1 Axle	Load 2 Axle	Load 3 Axle	Load 4 Axle	Load 5 Axle
Gross vehicle weight	0.371	0.653	0.257	0.616	0.684

## Data Availability

Not applicable.
